# To Get Lean

**Published:** 1870-07

**Authors:** 


### TO GET LEAN.

If a man is fat or lean and feels well, having all the bodily functions acting regularly, with sound sleep and no discomfort after eating, he should by all means let himself alone. Most persons want to have a little more flesh; want to weigh more; a few want to weigh less.

Some, in the effort to increase their weight, have eaten by rule and reason instead of being guided by their instinct, and have accomplished their object, with the addition of some chronic disease. Others, being too bulky for their pride, have paid for their fastidiousness by bringing on incurable maladies as a result of the too free use of vinegar or by chewing tea. In several cases Bright's disease of the kidneys has set in, and destroyed life.

Perhaps the safest way to get lean is to work hard and live mainly on fruits, bread and butter, berries, tomatoes, melons, and the like, using meat and vegetables only at dinner-time.

Steady exercise will diminish the weight of a common-sized man very rapidly. One hour and a half of gymnastic exercise will take two pounds off a good sized man. Some men weigh three or four pounds less in the morning than immediately after dinner. Persons are sometimes discouraged without cause because they weighed several pounds less than they expected, whereas, if they had weighed themselves in the same clothing, by the same scales, and at the same hour of the day, there would not have been a difference of an ounce.

Glass-blowers lose as much as five pounds in an hour. Men who perform unusual feats of walking have lost ten or a dozen pounds in twenty-four hours.

Vigorous health consists in a moderate amount of flesh. Too much flesh or fat is just as much disease as too little. In fat men the absorbents of the system do not work with sufficient activity; if they work too fast, they burn a man up, making him over lean and skinny. If a man is too lean and yet feels well, he thinks too much. Fat people sleep too much, are too lazy.

